# Comprehensive assessment of deceased donor kidneys with clinical characteristics, pre-implant biopsy histopathology and hypothermic mechanical perfusion parameters is highly predictive of delayed graft function

**DOI:** 10.1080/0886022X.2020.1752716

**Published:** 2020-04-25

**Authors:** Jin Zheng, Xiaojun Hu, Xiaoming Ding, Yang Li, Chenguang Ding, Puxun Tian, Heli Xiang, Xinshun Feng, Xiaoming Pan, Hang Yan, Jun Hou, Xiaohui Tian, Zunwei Liu, Xuzhen Wang, Wujun Xue

**Affiliations:** a Department of Renal Transplantation, Hospital of Nephrology, The First Affiliated Hospital of Medical College, Xi’an Jiaotong University, Xi’an, China; b Institute of Organ Transplant, Xi’an Jiaotong University, Xi’an, China

**Keywords:** Kidney transplant, clinical information, histopathology, HMP, acute tubular injury

## Abstract

**Background:**

Due to the current high demand for transplant tissue, an increasing proportion of kidney donors are considered extended criteria donors, which results in a higher incidence of delayed graft function (DGF) in organ recipients. Therefore, it is important to fully investigate the risk factors of DGF, and establish a prediction system to assess donor kidney quality before transplantation.

**Methods:**

A total of 333 donation after cardiac death kidney transplant recipients were included in this retrospective study. Both univariate and multivariate analyses were used to analyze the risk factors of DGF occurrence. Receiver operating characteristic (ROC) curves were used to analyze the predictive value of variables on DGF posttransplant.

**Results:**

The donor clinical scores, kidney histopathologic Remuzzi scores and hypothermic mechanical perfusion (HMP) parameters (flow and resistance index) were all correlated. 46 recipients developed DGF postoperatively, with an incidence of 13.8% (46/333). Multivariate logistic regression analysis of the kidney transplants revealed that the independent risk factors of DGF occurrence post-transplantation included donor score (OR = 1.12, 95% CI 1.06–1.19, *p* < 0.001), Remuzzi score (OR = 1.21, 95% CI 1.02–1.43, *p* = 0.029) and acute tubular injury (ATI) score (OR = 4.72, 95% CI 2.32–9.60, *p* < 0.001). Prediction of DGF with ROC curve showed that the area under the curve was increased to 0.89 when all variables (donor score, Remuzzi score, ATI score and HMP resistance index) were considered together.

**Conclusions:**

Combination of donor clinical information, kidney pre-implant histopathology and HMP parameters provide a more accurate prediction of DGF occurrence post-transplantation than any of the measures alone.

## Introduction

Aside from a living relative, donation after an individual’s death has become the only source of transplantable organs in China since 2015 [1], which only minimally alleviates the shortage of organ sources for ill patients. At present, more than 10,000 organ transplants are carried out in China every year, including kidney, liver, heart, lung, pancreas and small intestine. However, most deceased donation donors are considered extended criteria donors (ECD); while a patient may receive an organ, its quality and likelihood for full engraftment cannot be guaranteed. This results in a high incidence of graft failure, delayed recovery of graft function (DGF) and other related complications after kidney transplantation [2,3]. Therefore, the quality of donor kidneys and their suitability for transplantation need to be more accurately assessed in order to increase the likelihood of successful engraftment.

The quality assessment of a donor kidney is a comprehensive analysis [4,5] that includes clinical indicators of donor patients before donor kidney acquisition, the visual observation after donor kidney acquisition and the assessment of mechanical perfusion indicators during donor kidney maintenance [6,7]. The donor scoring system includes donor’s age, primary diseases, history of hypertension, pre-donation creatinine level, occurrence of cardiopulmonary resuscitation (CPR) and hypotension, all of which are closely related to DGF after kidney transplantation [4]. This scoring system based on DGF risk factor analysis has been shown to objectively evaluate the quality of donor kidneys [8,9,10].

Histopathological evaluation of the donor kidney *via* biopsy is an important component of the comprehensive evaluation, especially for ECD evaluation [4,5]. The pre-implantation biopsy is performed not only to evaluate the potential chronic changes in renal structure, but also to assess ischemic injury in the donated kidney [11]. Hypothermic mechanical perfusion (HMP) has been shown to mitigate DGF by removing residual renal microthrombi, dredging renal micro vessels and provide measures to assess renal function [6,7,12]. It provides a more optimal environment while the organ awaits transplantation, and can be exposed to variable temperatures and even treated with agents to minimize ischemia/reperfusion injury and decrease DGF [13].

It has been reported that DGF may increase the incidence of acute rejection after organ transplantation, increase hospitalization time and costs, affect the confidence of patients in recovery, contribute to an increased risk for developing chronic kidney disease and reduce the survival rate of transplanted kidney [14]. Therefore, it is important to investigate the risk factors of DGF and establish a comprehensive predictive system to assess donor kidney quality before transplantation on the occurrence of DGF. In this study, the correlation of donor patient parameters, kidney pre-implant pathology Remuzzi scores and HMP parameters were analyzed collectively, instead of individually, to enrich the comprehensive evaluation of donor kidney quality. This assessment can assist clinicians with more easily selecting the best donor organ for a given patient, even in the face of an organ shortage.

## Materials and methods

### Study cohort and ethics statement

We retrospectively studied the records of 181 donors and 333 recipients of a single kidney transplant at our center (Department of Kidney Transplant, the First Affiliated Hospital of Xi’an Jiaotong University) from January 2018 to September 2019. We excluded recipients that were less than 16 years old, re-transplantation patients, dual kidney and multi-organ transplants recipients. All patients underwent follow-up after transplantation and a database of relevant medical records was established. This cohort study was approved by the Institutional Review Board/Ethics of the First Affiliated Hospital of Xi’an Jiaotong University and was conducted in accordance with the principles of Declaration of Helsinki. No organs were obtained from prisoners in this study. Organs were obtained by the Organ Procurement Organization (OPO) of the First Affiliated Hospital of Xi’an Jiaotong University and were allocated by China Organ Transplant Response System (Cotrs).

### Data collection

Donor individual characteristics were collected including: age, sex, cause of death, serum creatinine (sCr) levels prior to organ recovery, history of hypertension, incidence of CPR and hypotension duration, organs cold ischemia time and warm ischemia time and ECD. Recipient characteristics at the time of transplant including: age, sex, number of previous kidney transplants, current level of panel reactive antibodies, number of human leukocyte antigen mismatches, DGF and recipients following up time.

### The donor scoring system

The donor scoring system included the donor’s age, primary disease, sCr levels prior to organ recovery, history of hypertension, CPR incidence and hypotension duration. The value of donor clinical scores in predicting graft performance was previously developed and validated from a thousand-patient cohort at our center [15]. Supplemental Table S1 shows the cutoffs used by the different histologic scoring systems.

### Machine perfusion

All donation after cardiac death (DCD) kidneys included in our study were perfused *in situ* and preserved by an HMP device (LifePort, Organ Recovery Systems). The perfusion pressure was initially set at 30–40 mmHg, and stabilizes after 15 min of perfusion. After a half-hour, if the flow was 140 mL/min, pressure was decreased to maintain 100–140 mL/min. Terminal pressure (P), flow (F) and resistance index (RI) were recorded at the end of perfusion, just before the transplantation.

### Pre-implantation biopsy evaluation

Pre-implantation biopsies were performed by the transplant surgeon using a 16G Bard needle. Two biopsies were obtained for each donation kidney. One tissue was embedded in optimum cutting temperature compound for immunofluorescence staining including IgA, IgM, IgG, C3, C1q, fibrin-related antigen. The other biopsy was fixed in formaldehyde, embedded in paraffin, sectioned and stained for hematoxylin and eosin, periodic acid-Schiff’s, Masson’s trichrome and silver methenamine. Light microscopy was performed, and Remuzzi’s method [16] was used to evaluate chronic histopathological changes in the donor kidney, and acute tubular injury (ATI) in the donor kidney was also assessed. Based on Remuzzi, the donor renal glomerulosclerosis, tubular atrophy, interstitial fibrosis and arterial lumen stenosis were each assessed by a pathologist as 0–3 points according to the degree of lesion. Supplemental Table S2 is a summary of the scores obtained in this study. All biopsies were performed pre-implantation, but the histopathologic diagnosis was determined after transplantation in order to avoid potential selection bias based on histopathological findings.

### Immunosuppression

All recipients were given a triple immunosuppressive regimen with calcineurin inhibitors (CNIs), enteric-coated mycophenolate sodium (EC-MPS; Myfortic, Novartis Pharma, Basel, Switzerland) and prednisone. The CNIs included cyclosporine A (CsA; Sandimmun Optoral, Novartis Pharma, Nuremberg, Germany) and tacrolimus (TAC; Prograf, Astellas Pharma, Deerfield, IL, USA). The initial dosages of CsA, TAC, EC-MPS and prednisone were 4.0–4.5 mg/kg/day, 0.06–0.08 mg/kg/day, 1080–1440 mg/day and 10–20 mg/day, respectively. All the recipients were treated with rabbit anti-thymocyte globulin (rATG; thymoglobulin, Genzyme Ireland, Waterford, Ireland) at a dosage of 1.25–1.50 mg/kg/day as induction therapy during the surgery, and a total of 4–6 days after kidney transplantation.

### Definitions

DGF was defined as dialysis required in the first 7 days after transplantation. Recipients receiving dialysis during the first week after renal transplantation for the reasons of acute rejection, or surgical complication were not regarded as DGF. ECDs were defined as donors aged 60 years and older, or those aged 50–59 years with at least two of the following conditions: cerebrovascular cause of death, terminal creatinine >1.5 mg/dL and/or hypertension.

### Statistical analysis

Data were analyzed by SPSS® version 17.0. The results are expressed as numerical values and percentages for categorical variables and as mean ± SD for continuous variables, unless otherwise stated. Spearman’s rho correlation was used to test the correlations among donor clinical score, biopsy pathology score and HMP parameters. Correlation is significant at the 0.05 level (2-tailed). Univariate and multivariate analysis were used to analyze the risk factors of DGF occurrence. Receiver operating characteristic (ROC) curves were used to compare the predictive value of variables on DGF post-transplant. *p* < 0.05 was considered statistically significant.

## Results

### Cohort description

All recipients in our cohort received DCD organs. The study covered 181 cases of donation, including 99 cases of standard criteria donation (54.7%) and 82 cases of ECD (45.3%). The baseline information on the donors and recipients is summarized in Table 1. The mean donor age was 50.8 ± 12.5 years (range 16–73 years); 48 donors (26.5%) were ≥60 years of age. The main primary diseases of donors were cerebral hemorrhage (45.3%) and trauma (43.1%). The mean terminal sCr concentration of donors before procurement was 113.0 ± 78.7 µmol/L. The mean warm and cold ischemia time were 5.1 ± 2.2 min and 8.7 ± 3.4 h, respectively. Cases having a history of hypertension were reported in 115 (63.5%) donors, and 23(12.7%) had received CPR. The cohort consisted of 333 recipients in whom grafts were adequately biopsied in the operating room pre-transplant. The other 29 recipients were excluded because of a lack of HMP parameters (18 cases) or pre-implantation biopsy (10 cases), or the need for graft excision one day post-transplant (one case). The mean recipient age was 36.2 ± 9.3 years (range 12–65). All recipients received their first allograft and most were not sensitized. DGF occurred in 46 (13.8%) cases. The mean follow-up time after transplantation was 297.8 ± 110.1 days, with all patients having a minimal follow-up time of 3 months.

**Table 1.** Donor and recipient characteristics.

Parameter	
Donor characteristics	*n* = 181
Age	50.8 ± 12.5
Male/female ratio	136/45
BMI	23.1 ± 2.9
Primary disease	
Trauma	78 (43.1%)
Cerebral hemorrhage	82 (45.3%)
Hypoxic encephalopathy	11 (6.1%)
Tumor	4 (2.2%)
Others	6 (3.3%)
sCr(µmol/L)	113.0 ± 78.7
BUN（mmol/L)	8.6 ± 4.9
Urine volume(ml/h)	186.8 ± 56.8
Warm ischemia time (min)	5.1 ± 2.2
Cold ischemia time (h)	8.7 ± 3.4
Hypertension	115 (63.5%)
CPR	23 (12.7%)
ECD	82 (45.3%)
Recipient characteristics	*n* = 333
Age	36.2 ± 9.3
Male/female ratio	237/96
HLA mismatches (Res M)	1.9 ± 0.8
PRA	44 (13.2%)
DGF	46 (13.8%)
Following up time (days)	297.8 ± 110.1

HLA: human leucocyte antigen; sCr: serum creatinine; BUN: blood urea nitrogen; CPR: Cardio pulmonary resuscitation; ECD: extended criteria donation; PRA: panel reactive antibody.

### Distribution of donor clinical score, biopsy pathology score and HMP parameters

**Figure 1.**
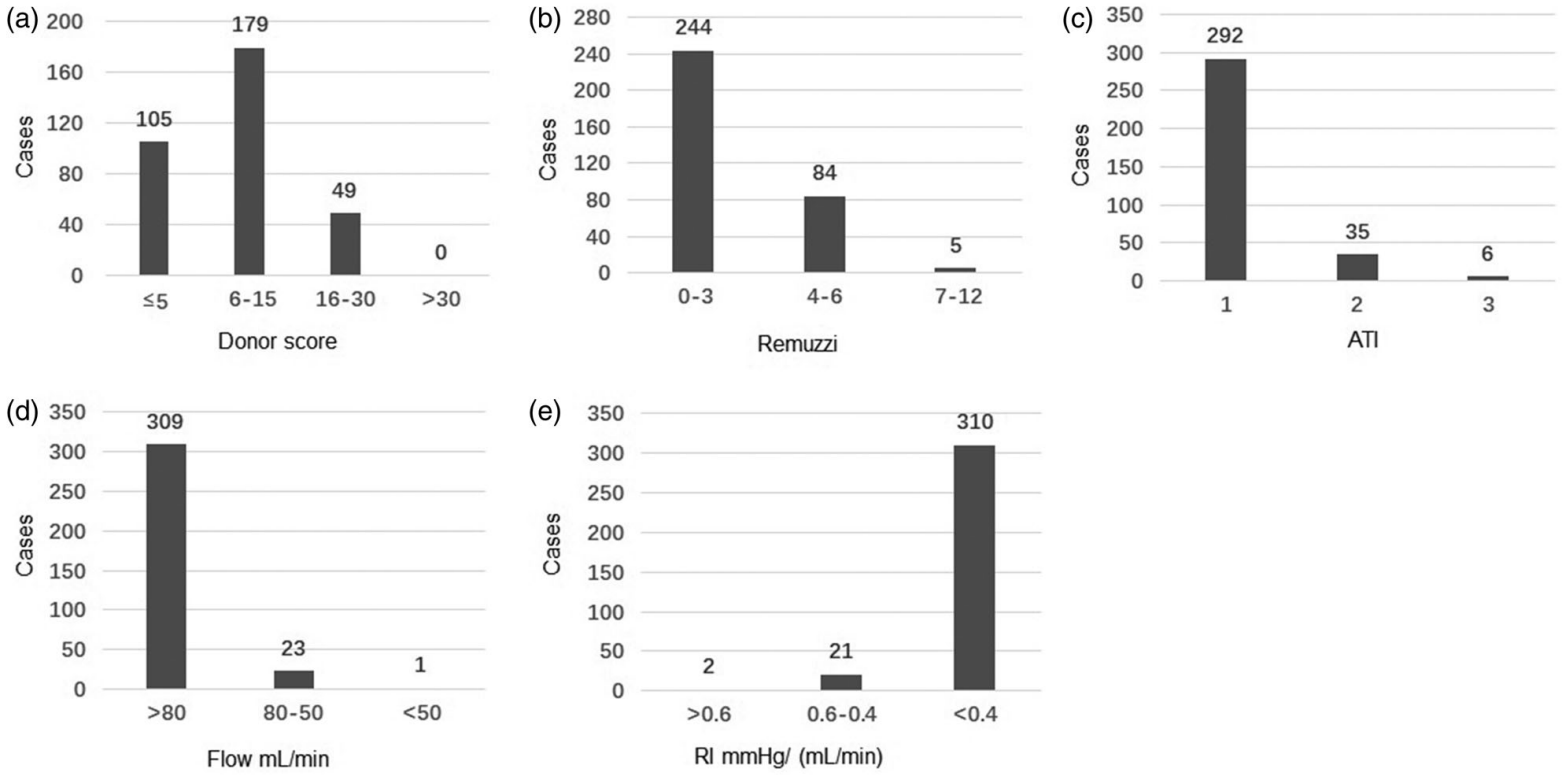
Distribution of the donor clinical scores, biopsy pathology scores and HMP parameters. (a) Distribution of donor clinical score; (b) Distribution of donor kidney Remuzzi score; (c) Distribution of donor kidney ATI score; (d) Distribution of donor kidney HMP flow parameter; (e) Distribution of donor kidney HMP RI parameter.

In 333 donors’ kidneys, clinical scores were distributed as follows: 105 cases (31.5%) were less than or equal to 5; 179 cases (53.8%) were in the range of 6 and 15; 49 cases (14.7%) were in the range of 16 and 30; the highest score was 27 (Figure 1(a)).

The distribution of graft biopsy pathology based on the Remuzzi score is shown in Figure 1(b). 244 grafts (70.2%) were scored 0–3, 84 grafts (25.2%) were scored 4–6 and only 5 grafts (1.5%) were in the range of 7 and 12 (the highest score was 8). For ATI, 292 grafts (87.7%) were considered mildly impaired, 35 grafts (10.5%) were moderately injured and 6 grafts (1.2%) had severe damage (Figure 1(c)).

Indices of flow and resistance based on HMP parameters is shown in Figure 1(d,e). Flow parameters of most grafts (309 cases, 92.8%) were more than 80 mL/min, and the lowest flow parameter was 48 mL/min. The resistance index of most cases (310/333; 93.1%) was less than 0.4 mmHg/(mL/min), and the highest resistance index was 0.70 mmHg/(mL/min).

### Correlations among donor score, Remuzzi score and HMP parameters

Spearman’s rho correlation was used to test the correlations among the donor score, Remuzzi score and HMP parameters. Donor score is significantly correlated with HMP flow or HMP RI (*p* < 0.001) (Table 2). A strong correlation also exists between either the donor score or HMP parameters and the Remuzzi score of donor kidneys, as shown in Table 2 (p < 0.001). However, no correlation was found between ATI and HMP parameters (*p* > 0.05) (Table 2).

**Table 2.** Correlation of donor scores, Remuzzi scores and HMP parameters.

Correlation of biopsy pathology and HMP parameter							
		Flow (mL/min)	Resistant index (mmHg/(mL/min))			Flow (mL/min)	Resistant index (mmHg/(mL/min))
Donor clinical score	Correlation coefficient	−0.183**	0.248**	Remuzzi	Correlation coefficient	−0.380**	0.356**
	Sig. (2-tailed)	0.001	<0.001		Sig. (2-tailed)	<0.001	<0.001

Correlation of donor clinical score and biopsy pathology score						
		Flow (mL/min)	Resistant index (mmHg/(mL/min)			Remuzzi
ATI	Correlation coefficient	0.016	0.053	Donor clinical score	Correlation coefficient	0.253**
	Sig. (2-tailed)	0.765	0.335		Sig. (2-tailed)	<0.001

**Correlation is significant at the 0.01 level (2-tailed).

### Risk factors of DGF

Univariate analyses for the risk factors of DGF showed that patients who received a kidney from an individual with a high donor score had a higher risk of developing DGF (OR:1.17, 95% CI: 1.11–1.24, *p* < 0.001), especially if there was a history of hypertension (OR: 1.18, 95% CI: 1.09–1.28, *p* < 0.001) or sCr before procurement (OR: 1.01, 95% CI: 1.01–1.02, *p* < 0.001). For histopathologic evaluation of pre-implantation biopsy, Remuzzi score (OR: 1.33, 95% CI: 1.15–1.53, *p* < 0.001) was also associated with a higher risk of DGF. Arteriole narrow (AN) score was also associated with DGF occurrence (OR: 1.82, 95% CI: 1.18–2.81, *p* = 0.007). ATI score was highly predictive for DGF (OR: 5.38, 95% CI: 2.85–10.18, *p* < 0.001). The HMP flow index and RI were also correlated with development of DGF after kidney transplantation, especially RI ≥ 0.3 mmHg/(mL/min) (OR: 2.97, 95% CI: 1.54–5.72, *p* = 0.001) (Table 3). In multivariate logistic regression analyses, donor score, Remuzzi score and ATI still were independent risk factors for DGF occurrence as shown in Table 4, especially ATI (OR: 4.72, 95% CI: 2.32–9.60, *p* < 0.001). On the other hand, history of hypertension, sCr before procurement, AN and HMP parameters were not independent risk factors of DGF occurrence (Table 4).

**Table 3.** Univariate analysis for risk factors of DGF (*N* = 333).

Variables	Odds ratios	95% Confidence interval for mean		*p*
		Lower bound	Upper bound	
Donor score	1.17	1.11	1.24	<0.001
Hypertension (mmHg)	1.18	1.09	1.28	<0.001
sCr (µmol/L)	1.01	1.01	1.02	<0.001
Remuzzi	1.33	1.15	1.53	<0.001
AN	1.82	1.18	2.81	0.007
ATI	5.38	2.85	10.18	<0.001
Flow (mL/min)	0.98	0.96	1.00	0.024
RI ≥ 0.3 (mmHg/(mL/min))	2.97	1.54	5.72	0.001

Logistic regressions were performed, odds ratios and the 95% confidence intervals were reported.

sCr: serum creatinine; AN: Arteriole Narrow; ATI: Acute tubular atrophy; RI: resistance index.

**Table 4.** Multivariate analysis for risk factors of DGF (*N* = 333).

Variables	Odds ratios	95% Confidence interval for mean		*p*
		Lower bound	Upper bound	
Donor score	1.12	1.06	1.19	<0.001
Remuzzi	1.21	1.02	1.43	0.029
ATI	4.72	2.32	9.60	<0.001
R1 ≥ 0.3 (mmHg/(mL/min))	1.84	0.85	4.01	0.124

Logistic regression were performed, odds ratios and the 95% confidence intervals were reported.

sCr: serum creatinine; AN: Arteriole Narrow; ATI: Acute tubular atrophy; F1: terminal flow; R1: terminal resistance.

### Predictive value of the composite parameters for DGF

**Figure 2.**
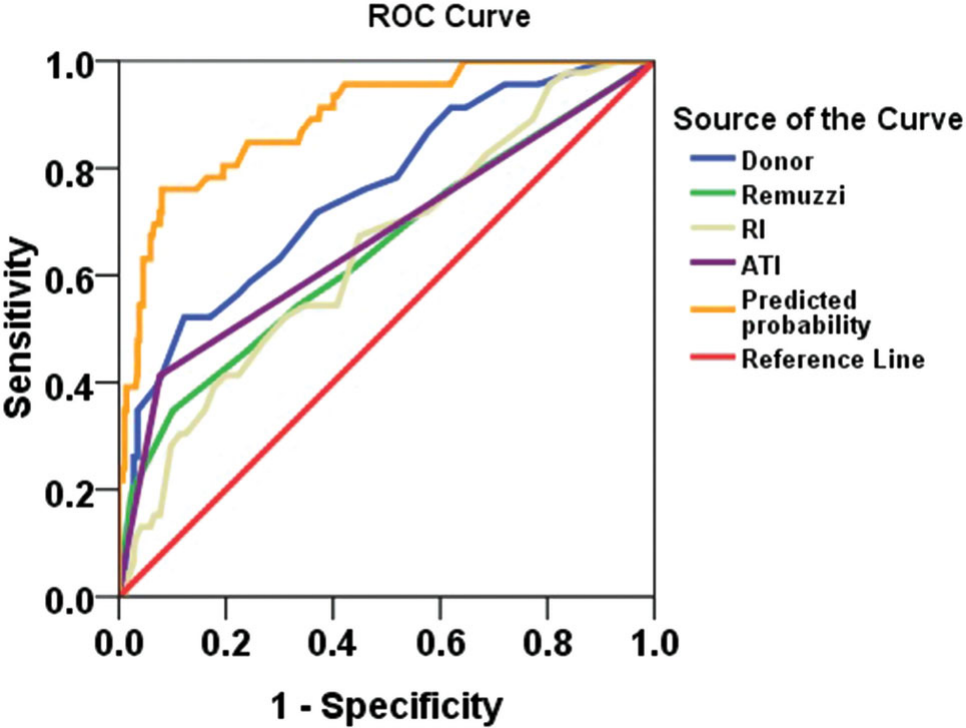
Receiver operating characteristic (ROC) curves for predicting DGF post-transplant. ATI: acute tubular injury score; RI: resistance index; Predicted probability: fitting value of all test variables.

Co-evaluation of DGF occurrence was based on donor clinical status, biopsy histopathology and HMP parameters by ROC curve. The ROC analysis showed the calculated area under the curve (AUC) of the five evaluated variables were lower than 80% (donor score, AUC = 0.75; Remuzzi score, AUC = 0.65; RI, AUC = 0.65; ATI, AUC = 0.67) (Table 5). However, the AUC was increased to 0.89 when all variables were fitting together as shown in Figure 2. The sensitivity and specificity of predicting DGF were 0.804 and 0.805, respectively.

**Table 5.** Receiver-operating characteristics (ROC) curves for clinical, histopathological and HMP parameters as predictors of DGF post-transplant.

Area under the curve					
Test result variable(s)	Area	Std. Error^a^	Asymptotic Sig.^b^	Asymptotic 95% confidence interval	
				Lower bound	Upper bound
Donor score	0.75	0.039	<0.001	0.68	0.83
Remuzzi	0.65	0.048	0.001	0.55	0.74
RI (mmHg/(mL/min)	0.65	0.044	0.002	0.56	0.73
ATI	0.67	0.049	<0.001	0.57	0.76
Predicted probability	0.89	0.025	<0.001	0.84	0.94

The test result variable(s): Donor score, Remuzzi, RI, ATI has at least one tie between the positive actual state group and the negative actual state group. Statistics may be biased.

^a^Under the nonparametric assumption.

^b^Null hypothesis: true area = 0.5.

## Discussion

The incidence of DGF is on the rise because of an increasing use of marginal kidneys in an era of organ shortage [17]. Risk factors for DGF are numerous and stem from multiple sources in the transplant chain starting from the donor to its final allocation in the recipient [18,19]. DGF refers to the acute kidney injury which caused by CPR or hypotension during the donation process and could be reflected by high sCr before donation [17,20]. Our data showed that donor score, Remuzzi score and ATI all were the independence risk factor of DGF. This indicates that both acute and chronic lesions of donation kidney play an important role in the occurrence of DGF, which consistent with other studies [5].

Clinical information and laboratory results of potential donors are important for initial assessment of the quality of an organ. The Kidney Donor Profile Index (KDPI) is a score that estimates the risk of graft failure [21,22]. On the base of KDPI score combined with donors’ actual situation in China, we consider donor age, cerebrovascular cause of death, history of hypertension, sCr before kidney procurement, hypotension and CPR incidence as a suitable donor evaluation system [15]. The pre-implantation biopsy is frequently performed and serves as another important tool for evaluating the kidney lesions, particularly in cases of ECD [5,23]. To better quantitate histopathologic features, the Remuzzi scoring system was adapted to assess chronic lesions of donation kidneys. HMP was usually used to remove residual thrombi from the microcirculation, in order to improve early function and graft survival [16,24]. Furthermore, HMP enables the assessment of graft viability and quality pre-implantation [25] through tracking measures of perfusate flow rate and vascular resistance [26,27].

It was found that donor score, Remuzzi score and HMP parameters were correlated with each other in our study. In particular, donor score, hypertension history, sCr before procurement, Remuzzi score, AN, ATI and HMP RI ≥3 were risk factors of DGF occurrence. Furthermore, donor score, Remuzzi score and ATI were independent risk factors for DGF occurrence post-transplantation, especially ATI. Combining donor score, Remuzzi score, ATI and HMP RI ≥3 together provides the most accurate prediction of DGF.

Clinical information, biopsy histopathology and HMP parameters were all indicated for risk assessment of donated kidneys, implying some kind of relationship among them. Kidneys from donors that are elderly, hypertensive or those with renal dysfunction could have chronic lesions such as glomerulosclerosis, tubular atrophy, interstitial fibrosis and arterial sclerosis. In the present study, all chronic lesions were reflected by the clinical characteristics of donors, so that donors’ clinical scores correlated with kidney biopsy histopathology scores. These lesions would also disturb the microcirculation and influence HMP parameters. These phenomena were identified in the current study by the correlation of both donor clinical score and histopathology score with HMP parameters (especially RI).

We found that donor score, donation kidney Remuzzi score and HMP parameters combined together can not only evaluate the quality of donor kidney, but also more accurately predict the occurrence of DGF. In our study, the AUC predicted DGF was increased to 0.89 when fitting the key variables (Donor score, Remuzzi score, ATI and RI) together. The sensitivity and specificity of predicting DGF were 0.804 and 0.805, respectively. These data illustrate the importance of a more comprehensive evaluation of an organ. No one has fully analyzed whether these parameters can be combined to improve the quality of a donated kidney. In the past, donated organs assessed for use or discard based solely on pathological results had been denied by most transplant doctors [4]. At present, more transplant centers have begun to conduct comprehensive assessment of the donor kidney more thoroughly, so as to ensure a more safe and effective use of the donor kidney. Ideally, kidneys will be neither wasted nor used in poor condition. Of course, it is difficult to achieve this, and additional in-depth evaluation systems, such as molecular markers, irrigation fluid culture, susceptibility-weighted imaging and other information, need to be further investigated to improve quality of organ transplantation [28–31].

Interestingly, there was no correlation between ATI and HMP parameters. we speculate that the main lesion site of acute kidney injury was the renal tubule, which has little influence on microcirculation. In addition, there was no statistical correlation between glomerular sclerosis (GS) and HMP parameters (data not shown). One possibility is that the glomerulosclerosis ratio of most cases in our study was lower than 20%, which would not overtly affect HMP parameters. Another other reason for a lack of correlation in these parameters might be related to the technical and practical limitations of fine needle puncture and biopsy sampling.

Diabetes can lead to hyaline arterioles and hypertrophy of the basement membrane. In the kidney, the main manifestations are capillary glomerulosclerosis (Kimmelstiel–Wilson nodules) and tubular basement membrane hypertrophy and papillary necrosis, which severely affects kidney function. However, the donor history of diabetes was very rare in our study (4/333). Therefore, diabetic donors were classified as other cases and not listed separately for analysis. Trauma (43.1%) and cerebral hemorrhage (45.3%) were the major causes of death in our study, accounting for 88.4% of total cases. However, diabetes rarely causes cerebral hemorrhage, and is also rare in the trauma cases.

In conclusion, our study provides a new way to comprehensively evaluate donor kidneys by combining clinical characteristics, biopsy histopathology features and HMP parameters. Donor score, Remuzzi score and HMP parameters were correlated with each other in our study. In particular, donor score, hypertension history, sCr before procurement, Remuzzi score, AN, ATI and HMP RI ≥3 were risk factors of DGF occurrence. Furthermore, donor score, Remuzzi score and ATI were independent risk factors for DGF occurrence post-transplantation. Importantly, our analysis shows that donor score, donation kidney Remuzzi score and HMP parameters combined together can not only evaluate the quality of donor kidney, but also more accurately predict the occurrence of DGF.

## Supplementary Material

Supplemental Material

## ABBREVIATIONS

AH: arteriolar hyalinosis
AN: arteriole narrow
ATI: acute tubular injury
AUC: area under the curve
CPR: cardiopulmonary resuscitation
DCD: donation after cardiac death
DD: deceased donation
DGF: delayed recovery of graft function
ECD: extended criteria donor
GS: glomerular sclerosis
HLA: human leukocyte antigen
HMP: hypothermic machine perfusion
IF: interstitial fibrosis
MAPI: Maryland aggregate pathology index
PGF: peri-glomerular fibrosis
PNF: primary non-function
PRA: panel reactive antibodies
ROC: receiver operating characteristic curve
sCr: serum creatinine
TA: tubular atrophy
WLR: arterial well to lumen ratio
