# Supplementary material for: Comprehensive assessment of deceased donor kidneys with clinical characteristics, pre-implant biopsy histopathology and hypothermic mechanical perfusion parameters is highly predictive of delayed graft function
Source: Ren Fail. 2020 Apr 25;42(1):369–76. doi: 10.1080/0886022X.2020.1752716 (PMC7241463; doi:10.1080/0886022X.2020.1752716)
Supplement: Supplemental Material [file IRNF_A_1752716_SM7570.pdf]

Table S1. Clinical scoring criteria for donors

Item		Score	Item	Score
Age (Years)			Primary disease	
16-39		0	trauma	0
40-49		1	Cerebral hemorrhage	3
50-64		2	hypoxic ischemic encephalopathy	6
≥65		3	Others	3
Hypotension			History of hypertension (Years)	
None		0	None	0
SBP<80mmHg	SBP<50mmHg		0-4	5
<1h	<10min	5	5-9	6
>1h	>10min	8	≥10	7
sCr(μmol/L)			Time of CRP (min)	
<177		0	None	0
177-265		7	0-9	5
266-442		8	10-29	7
≥443		17	≥30	8

Table S2. Remuzzi histopathological evaluation system of donor kidney biopsy

**Remuzzi histopathological evaluation system of donor kidney biopsy**	
Variables	Points and contains
Glomerular global sclerosis<br>(Based on three sections (the first, middle, and last sections, if available); the number of globally sclerosed glomeruli expressed as a percentage)	0. none globally sclerosed<br>1+, <20% global glomerulosclerosis<br>2+, 20 to 50% global glomerulosclerosis<br>3+, >50% global glomerulosclerosis
Tubular atrophy	0, absent<br>1+, <20% of tubule affected<br>2+, 20 to 50% of tubule affected<br>3+, >50% of tubule affected
Interstitial fibrosis	0, absent<br>1+, <20% of renal tissue replaced by fibrous connective tissue<br>2+, 20 to 50% of renal tissue replaced by fibrous connective tissue<br>3+, >50% of renal tissue replaced by fibrous connective tissue
Arterial and arteriolar narrowing<br>(For the vascular lesions, if the changes are focal, the most severe lesion present gives the final grade)	0, absent<br>1+, increased wall thickness but to a degree that is less than the diameter of the lumen<br>2+, wall thickness that is equal or slightly greater to the diameter of the lumen<br>3+, wall thickness that far exceeds the diameter of the lumen with extreme luminal narrowing or occlusion
Final grade<br>(The final grade can range from 0 to a total of 12)	0 to 3 mild OK for single transplant<br>4 to 6 moderate OK for double transplant<br>7 to 12 severe should not be transplanted
